# The Role of BTBD9 in Striatum and Restless Legs Syndrome

**DOI:** 10.1523/ENEURO.0277-19.2019

**Published:** 2019-10-08

**Authors:** Shangru Lyu, Hong Xing, Mark P. DeAndrade, Yuning Liu, Pablo D. Perez, Fumiaki Yokoi, Marcelo Febo, Arthur S. Walters, Yuqing Li

**Affiliations:** 1 Norman Fixel Institute for Neurological Diseases, Department of Neurology, College of Medicine, University of Florida Gainesville, FL 32610; 2 Department of Psychiatry, College of Medicine, University of Florida Gainesville, FL 32610; 3 Division of Sleep Medicine, Vanderbilt University Medical Center, Nashville, TN

**Keywords:** BTBD9, cholinergic interneuron, medium spiny neuron, Restless legs syndrome, sleep, striatum

## Abstract

Restless legs syndrome (RLS) is a sensory-motor neurological disorder characterized by uncomfortable sensations in the extremities, generally at night, which is often relieved by movements. Genome-wide association studies (GWAS) have identified mutations in *BTBD9* conferring a higher risk of RLS. Knockout of the *BTBD9* homolog in mice (*Btbd9*) and fly results in motor restlessness and sleep disruption. Clinical studies have found RLS patients have structural and functional abnormalities in the striatum; however, whether and how striatal pathology contributes to the pathogenesis of RLS is not known. Here, we used fMRI to map regions of altered synaptic activity in basal ganglia of systematic *Btbd9* knock-out (KO) mice. We further dissected striatal circuits using patch-clamp electrophysiological recordings in brain slices. Two different mouse models were generated to test the effect of specific knockout of *Btbd9* in either striatal medium spiny neurons (MSNs) or cholinergic interneurons (ChIs) using the electrophysiological recording, motor and sensory behavioral tests. We found that *Btbd9* KO mice showed enhanced neural activity in the striatum, increased postsynaptic currents in the MSNs, and decreased excitability of the striatal ChIs. Knocking out *Btbd9* specifically in the striatal MSNs, but not the ChIs, led to rest-phase specific motor restlessness, sleep disturbance, and increased thermal sensation in mice, which are consistent with results obtained from the *Btbd9* KO mice. Our data establish the role of *Btbd9* in regulating the activity of striatal neurons. Increased activity of the striatal MSNs, possibly through modulation by the striatal ChIs, contributes to the pathogenesis of RLS.

## Significance Statement

Restless legs syndrome (RLS) is a common movement disorder affecting up to 10% of the population and its pathophysiology is largely unknown. Brain imaging studies have shown striatal involvement. However, whether and how striatal pathology contributes to the pathogenesis of RLS is not known. Polymorphisms in the *BTBD9* gene are associated with RLS. *Btbd9* complete knock-out (KO) mice have RLS-like phenotypes. With a combination of methods including fMRI, brain slice electrophysiology, cell type-specific KO, and behavioral tests, we demonstrate the importance of the striatum, especially the MSNs, in the pathogenesis of RLS. Our results also suggest a novel mechanism that can explain the effectiveness of dopaminergic drugs for the treatment of RLS patients.

## Introduction

Restless legs syndrome (RLS) is a sensorimotor neurologic disease affecting up to 10% of the general population (Garcia Borreguero et al., 2017). Characteristic symptoms of RLS include an urge for patients to move their legs often accompanied by, or felt to be caused by, uncomfortable sensations in the legs (Lanza and Ferri, 2019). The symptoms of RLS generally occur or worsen at rest or inactivity in the evening, which can be at least partially relieved by movements (Trenkwalder et al., 2018). Previous studies have emphasized the major role of iron in the disease (Connor et al., 2017). In addition, one of the primary medications for the disease is D_2_/D_3_ dopamine (DA) agonists (Garcia-Borreguero and Cano-Pumarega, 2017), whereas refractory RLS can be treated with opioids (Silber et al., 2018).

To date, no neurodegeneration has been found in RLS patients. However, emerging studies suggest that changes in the striatum may underlie the pathogenesis of RLS (Earley et al., 2017; Lanza et al., 2017; Rizzo et al., 2017). The striatum, which is comprised of the caudate and putamen, serves as the first recipient for most of the excitatory input from the cortex and thalamus to the basal ganglia (Haber, 2016). Approximately 95% of striatal neurons are GABAergic medium spiny neurons (MSNs), which are traditionally subdivided into two subtypes by DA receptor expression (Purves et al., 2001b). Generally, D_1_ DA receptor (D_1_R)-expressing MSNs function in the direct pathway and are thought to facilitate wanted movements and pronociceptive effects (Soares-Cunha et al., 2016). In contrast, MSNs in the indirect pathway mainly express D_2_ DA receptors (D_2_Rs). These MSNs are likely to be involved in the suppression of unwanted movement and the generation of antinociceptive effects (Soares-Cunha et al., 2016). Additionally, 1–2% of striatal neurons are cholinergic interneurons (ChIs; Zucca et al., 2018). MSNs regulate the activity of ChIs through GABA and endogenous opioids (Lim et al., 2014), while ChIs influence the activity of MSNs through M1, M4, and possibly nicotinic acetylcholine (ACh) receptors on MSNs (Bordia et al., 2016). Previous clinical studies have shown decreased D_2_R expression but increased phosphorylated TH in the putamen (Connor et al., 2009). Brain imaging studies show decreased striatal DA transporter (DAT; Earley et al., 2011) and D_2_R binding potential (Michaud et al., 2002; Earley et al., 2013). Despite these observed alterations of the striatal dopaminergic system associated with RLS, the exact functions of the striatum, especially MSNs, in the disease development are largely unknown.

Genome-wide association studies (GWAS) have implicated up to 19 risk loci, including *BTBD9*, as genetic risk factors of RLS (Schormair et al., 2017; Jiménez-Jiménez et al., 2018). *BTBD9* codes for a protein belonging to the BTB (POZ) protein family, which modulates transcription, cytoskeletal arrangement, ion conductance and protein ubiquitination (Stogios and Privé, 2004; Stogios et al., 2005). An alteration in hippocampal synaptic plasticity and neurotransmission has been found in *Btbd9* knock-out (KO) mice (DeAndrade et al., 2012b). Loss of the BTBD9 homolog in *Drosophila melanogaster* results in increased motor activity, decreased DA levels, and disrupted sleep patterns (Freeman et al., 2012). Similarly, the systematic *Btbd9* KO mice showed motor restlessness, thermal hypersensitivity, and a disruption in sleep structure (DeAndrade et al., 2012a). Therefore, the systematic *Btbd9* KO can be considered as a valuable disease mouse model to study the pathophysiology of RLS (DeAndrade et al., 2012a; Allen et al., 2017).

To elucidate the impact of striatal neurons on the generation of RLS-like phenotypes, we performed *in vivo* manganese-enhanced MRI (MEMRI) with the systematic *Btbd9* KO mice and mapped the functional neural activity in the basal ganglia circuits. We next conducted electrophysiological recordings to observe both intrinsic and spontaneous firing activity of MSNs and ChIs in the striatum. Moreover, we selectively deleted *Btbd9* in either striatal MSNs or ChIs and conducted behavioral studies.

## Materials and Methods

### Mice

#### The generation of the systematic *Btbd9* KO mice

The homozygous *Btbd9* KO male mice used in MRI imaging were generated as described previously (DeAndrade et al., 2012a). The systematic *Btbd9* KO mice used in the electrophysiological recording were generated from a line of *Btbd9 loxP* mice imported from the European Mouse Mutant Archive (EMMA; ID: 05554). In this line, the fourth exon of the *Btbd9* gene was flanked by *loxP* sites (floxed). We first removed neomycin selection cassette by crossing with FLP mice (The Jackson Laboratory stock 126 no. 003946) to obtain *Btbd9 loxP* mice, which was then crossed with a general cre deletor to obtain *Btbd9* KO allele. Heterozygous *Btbd9* KO mice were interbred to produce experimental homozygous *Btbd9* KO mice and the wild-type (WT) littermate controls.

#### The generation of specific *Btbd9* KO mice

The MSNs-specific *Btbd9* KO mice (*Btbd9* sKO) were generated by breeding *Btbd9 loxP* mice with *Rgs9-cre* mice, in which the *cre* gene was inserted at the 3’ end of the *Rgs9* gene (Dang et al., 2006). Double heterozygous mice (*Rgs9-cre*±*Btbd9 loxP*±) were used for breeding with heterozygous (*Btbd9 loxP*±) or homozygous Btbd9 loxP mice (*Btbd9 loxP-/-*) to generate the conditional KO animals (*Rgs9-cre*±*Btbd9 loxP-/-*) and control groups, including WT littermates, animals only expressing *Rgs9-cre* (*Rgs9-cre*±) and animals only having loxP sites in one (*Btbd9 loxP*±) or both of the DNA strands (*Btbd9 loxP-/-*). PCR was used for genotyping the *Rgs9-cre* (forward: TGC TCA AAA ATT GTG TAC CTT TAG C; reverse: CAA CAC CCC ATT CGC TTT TTC CA) and the *loxP* sites (forward: ACA TCA CCC ATT ACT TAG AAC CTC; reverse: CAC AGC TAT TTC CTG TCA TTC TGG ACA).

The ChI-specific *Btbd9* KO mice (*Btbd9* ChKO) were generated by breeding *Btbd9 loxP* mice with *Chat-cre* mice (The Jackson Laboratory; stock 006410), in which the neo cassette had been removed by crossing with FLP mice. Breeding was conducted as outlined for *Btbd9* sKO above. PCR was used for genotyping the *Chat-cre* (forward: ATC TCC GGT ATT GAA ACT CCA GCG C; reverse: CAC TCA TGG AAA ATA GCG ATC). To confirm the specific deletion of *Btbd9* in the striatum, we dissected out brain regions following the protocol (Spijker, 2011) and PCR was conducted with primers specific for recombined locus (forward: AAG GCG CAT AAC GAT ACC ACG AT; reverse: TGG TGA TTC AAA TCT CCT TCC AAC ACA; Fig. 4*C*).[AQ3] Experimental mice were housed in standard mouse cages at 21°C under normal 12/12 h light/dark cycle (12 LD) condition. The protocol for the study received prior approval by the Institutional Animal Care and Use Committee, and all studies were conducted in accordance with the United States Public Health Service’s Policy on Humane Care and Use of Laboratory Animals.

#### Quantitative RT-PCR (qRT-PCR)

qRT-PCR was performed as described before (Yokoi et al., 2011) to determine whether exon 4 was deleted in mice after *cre-*mediated recombination. In brief, three *Btbd9* sKO and three control adult male mice were sacrificed, and several brain regions (striatum, cerebral cortex and cerebellum) were harvested and flash frozen in liquid nitrogen. RNA was extracted using an RNAeasy Mini kit (QIAGEN) according to the manufacturer’s instructions. Next, cDNA was made using SuperScript III reverse transcriptase (Invitrogen). PCR primers specific to *Btbd9* exons 4 and 5 (forward: GAC TCT TGT CTC CGG ATG CT; reverse: TCA CAA CCT GAG CCC CAT AC); β-actin (forward: CAC CCG CGA GCA CAG CTT CTT TG; reverse: AAT ACA GCC CGG GGA GCA TCG TC). The expression of *Btbd9* mRNA was measured and normalized by Bio-Rad CFX manager 3.1.

### MEMRI

#### MnCl_2_ (manganese chloride) pretreatment

MEMRI was performed as described previously (Perez et al., 2018; Zubcevic et al., 2018). Before the treatment, the animals used in the experiment were handled every two weeks and acclimatized to the investigator. Manganese (II) chloride tetrahydrate (Sigma-Aldrich Chemical Co.) was dissolved in distilled deionized water and sterilely filtered before administered intraperitoneally at a dose of 70 mg/kg/ml. After injections, mice were returned to their home cage and imaged after 20–24 h as previously reported (Perez et al., 2013).

### MRI

Images were collected by a 4.7 Tesla Magnex Scientific scanner under the control of Agilent Technologies VnmrJ 3.1 console software. A 38-mm quadrature transmit/receive radio frequency coil tuned to 200 MHz was used (Insight NeuroImaging Systems, LLC). Mice were anesthetized with 2.0% (0.1 l/min) delivered in 100% oxygen for 30–60 s. Then the level of isoflurane was maintained between 1.0% and 1.25% throughout the entire setup and imaging session, during which the respiratory rates were monitored continuously and sustained between 20 and 30 beats per minute by adjusting isoflurane levels between the range. Placed prone on custom-size plastic bed with a respiratory pad placed underneath the abdomen, body temperatures of the mice were maintained using a warm air recirculation system (SA Instruments, Inc.). The head and incisors of mice were secured on the front end of the plastic bed to minimize motion. The front half of the bed was aligned and clamped inside the quad RF coil and placed inside the isocenter of the scanner. Images were acquired at 4.7 Tesla using a T1-weighted spin echo pulse sequence with the following parameters: repetition time = 300 ms, echo time = 12 ms, the field of view = 19.2 × 19.2, slice thickness = 0.8 mm, 12 slices. Total scan time per mouse was 30 min.

### Electrophysiological recording

#### Slice preparation

Experiments were conducted as described previously (Pappas et al., 2015; Augustin et al., 2018). Recordings of MSNs were conducted with three *Btbd9* KO and five WT male littermates at an average age of four months. Recordings of ChIs were performed with four *Btbd9* KO and five WT male littermates with an average age of four months, or with three *Btbd9* ChKO and three control males with an average age of five months. Investigators who conducted the experiments were blind to the genotypes. Animals were sacrificed, and the brains were rapidly removed; 300 μm-thick coronal brain slices containing the dorsal striatum were cut in ice-cold, oxygenated cutting saline: 180 mM sucrose, 2.5 mM KCl, 1.25 mM NaH_2_PO_4_, 25 mM NaHCO_3_, 10 mM D-glucose, 1 mM CaCl_2_, 10 mM MgCl_2_, and 10 mM glucose with a Vibratome (Leica VT 1000s). Slices were recovered in a holding chamber for 30 min at 35°C with artificial CSF (ACSF). Final concentrations of ACSF: 126 mM NaCl, 2.5 mM KCl, 1.25 mM NaH_2_PO_4_, 25 mM NaHCO_3_, 2 mM MgCl_2_, 2 mM CaCl_2_, and 10 mM glucose, pH 7.3 with KOH, osmolality 290–300 mOsm. The slices were then incubated at room temperature.

#### Cell identification

The slices were placed in a recording chamber and continuously perfused with ACSF that was bubbled via 5% CO_2_ and 95% O_2_ at a rate of 1.5 ml/min while being visualized with an upright microscope (Zeiss) using a 40× water-immersion objective with infrared optics. MSNs were identified by the somatic size and basic membrane properties including input resistance, membrane capacitance, and time constant. ChIs were recognized based on morphology and size, as they are irregularly polygonal with large cell soma (>20 μm).

#### Cell-attached and whole-cell recordings

For MSNs, all experiments were recorded at 32°C by a dual automatic temperature controller (TC-344B). Cell-attached recording patch pipette (6–10 MΩ) contained following solutions: 125 mM K-gluconate, 8 mM NaCl, 10 mM HEPES, 2 mM MgATP, 0.3 mM NaGTP, and 0.2 mM EGTA (pH 7.25–7.3, osmolality 290–300 mOsm) and was used for voltage and current clamp recordings. Access resistances were < 30 MΩ. Spontaneous postsynaptic currents were recorded in ACSF. To minimize the contribution of GABA_A_ receptors, we held cells at −70 mV with an application of 50 µM picrotoxin solution, which can abolish the activation of GABA_A_ receptors. Next, at holding potential –65 mV, injection of depolarizing 50-pA current pulse of 300-ms duration evoked spike firing when the membrane potential reaches the firing threshold under current clamp configuration in a brain slice. This process was repeated at 10 increasingly depolarized potentials with incremental current steps (50 pA).

For ChIs, electrodes for cell-attached recordings were filled with a K-gluconate-based solution containing the following concentrations: 112.5 mM K-gluconate, 4 mM NaCl, 17.5 mM KCl, 0.5 CaCl_2_, 5 mM MgATP, 1 mM NaGTP, 5 mM EGTA, and 10 mM HEPES; with pH 7.2 (270–280 mOsm) and resistance of 5–10 MΩ. Positive pressure was applied to the patch electrode as it approached the ChIs. Suction was applied to the electrode to create a seal (>5 GΩ) between the recording pipette and cell membrane. Action potential current was recorded in a voltage-clamp mode that maintained an average of 0-pA holding current. After breaking through the cell membrane, cellular properties (capacitance, input resistance, and time constant) were recorded at a membrane potential of −70 mV. Electrode access resistance was maintained throughout at <30 MΩ. Resting membrane potential was recorded in current clamp mode. Action potential for current step recording was triggered using depolarizing currents steps of 300 ms.

Data acquirement and detection were the same as previously described (DeAndrade et al., 2012b). Recordings were made from targeted cells in the striatum using infrared differential interference contrast microscopy and an Axopatch 1D amplifier (Molecular Devices). Data were acquired using pClamp 10 software. Signals were filtered at 5 kHz, digitized at 10 kHz with a DigiData 1440 (Molecular Devices). Events were detected using the Mini Analysis Program (Synaptosoft) with parameters optimized for each cell and then visually confirmed before analysis. The peak amplitude, 10–90% rise time and the decay time constant were measured based on the average of all events aligned by the rising phase.

#### Immunofluorescence staining

*Rgs9-cre* mice were bred with GFP mice imported from The Jackson Laboratory (stock 007906) to obtain *Rgs9*-*cre* and GFP double heterozygous mice to map *Rgs9*-*cre-*positive neurons. As described previously (Dang et al., 2006), the mice were anesthetized and perfused with ice-cold 0.1 M PBS (pH 7.4) followed by 4% paraformaldehyde in 0.1 M PB (pH 7.4). The brains were soaked in 4% paraformaldehyde-PB at 4°C overnight and then incubated in 30% sucrose in 0.1 M PBS at 4°C until the brains sank to the bottom. The brains were frozen with dry-ice powder and cut coronally into 40-μm sections with a Histoslide 2000 sliding microtome (Reichert-Jung). Sections were sequentially rinsed 5 min each in 0.5% Triton X-100, 0.02 M PBS; 0.1% Triton X-100, 0.02 M PBS; 10 mM glycine in 0.1 M PBS for three times; 0.5-ml 2% gelatin in 0.1 M PBS; 10 mM glycine in 0.1 M PBS; and 0.1% BSA in 0.1 M PBS. Then tissues were incubated with the primary antibody, 1:50 goat anti-choline acetyltransferase (AB144P; Millipore), dissolved in 100 μl 1% BSA, 0.1 M PBS at 4°C overnight. The next day, tissues were washed for six times with 0.1% BSA, 0.1 M PBS, followed by incubation with secondary antibody, 1:200 Cy3-conjugated AffiniPure donkey anti-goat IgG (705-265-003; Jackson ImmunoResearch). After washing, the sections were mounted on glass slides with VECTASHIELD Antifade Mounting Medium (H-1400) and cover-slipped.

### Behavioral studies

#### Thirty-minute open field

Eight *Btbd9* sKO male mice and seven male littermates with an average age of 15 months, or seven *Btbd9* ChKO (three males, four female) and nine controls (five males, four female) with an average age of seven months, were used in the 30-min open field analysis as previously described (DeAndrade et al., 2012a). Briefly, each mouse was placed in the center of a VersaMax Legacy open field apparatus connected to a computerized Digiscan System (Accuscan Instruments, Inc.) and continuously monitored for 30 min. The apparatus contains infrared sensors along the walls that detect any breaks in the beams. Bright illumination (∼1 k lux at the center by a 60-W white bulb) was focused on the center of each field.

#### Wheel running

Eight *Btbd9* sKO mice (seven males, one female) and 13 control mice (eleven males, two females) with an average age of four months, or six *Btbd9* ChKO male mice and seven male littermates with an average age of two months, were maintained on a 12 LD cycle for 7 d. Wheel-running activity (DeAndrade et al., 2012a) was recorded as the number of wheel revolutions occurring during 5 min bins and analyzed using Lafayette Instrument Activity Wheel Monitor software. The activity from the last 4 d was included in the data analysis, grouped by light phase and dark phase.

#### Continuous open field

Seven male *Btbd9* sKO mice and five male littermates with an average age of five months or three *Btbd9* ChKO male mice and four male littermates with an average age of two months were used in the long-term open field analysis modified from 30-min open field test (Meneely et al., 2018). Each mouse was placed in the center of a VersaMax Legacy open field apparatus with enough corncob bedding, food, and water. Breaks in the beams were decoded by VERSDATA version 2.70-127E (AccuScan Instruments Inc.) into behavioral patterns. Batch 1 data for *Btbd9* ChKO mice were collected every 1 h. Other data were recorded every 15 min throughout the experiment. Data from the last 4 d were separated into light and dark phases, and the total distances during each phase were combined and coded as day 4–7, and night 4–7, respectively. The analysis was conducted based on all four periods in each phase. Separately, the total distances for each 15 min of the last 4 d were recoded for sleep analysis. If the total distance traveled in 15 min was 0, the mouse was considered as sleeping, and the data were coded as 0; otherwise, the mouse was considered as awake, and the data were coded as 1.

#### Tail flick test

Nine male *Btbd9* sKO mice and nine male littermates with an average age of eight months were tested for the perception of warm stimuli. Each mouse was placed in an acrylic restrainer with the distal end of its tail protruding on a metal surface maintained at 55°C. The timer was turned on once the tail touched the surface and immediately stopped when the mouse flicked its tail away from the heat. The latency to respond was limited to 90 s to prevent injury to the mouse.

#### Experimental design and statistical analysis

Images were processed and analyzed as previously reported (Perez et al., 2013). Mn^2+^ accumulation in active neurons produces signal intensity increases in T1 images. However, as this is a non-quantitative approach to measure activity and because there is scan-to-scan intensity variation independent of Mn^2+^, we normalized images based on their individual variance. Using this normalization approach, where surpassing a normalized threshold value of 1 indicates increased activity associated with Mn^2+^ administration, we have observed significant differences between Mn^2+^ administered and non-treated rodents. Image processing was conducted using ITK-SNAP (http://www.itksnap.org), and image math scripts were available on FSL (fslmaths; http://www.fmrib.ox.ac.uk/fsl/). Scans were aligned with a segmented atlas of the adult mouse brain using an automated affine linear registration tool from FSL (Jenkinson et al., 2012). Each scan was converted to a z value map through a voxel-wise normalization procedure. The mean signal intensity across the entire extracted brain volume (x¯) was subtracted from each voxel (xi) and then divided by the variance (σ). A pre-set threshold of z ≥ 1 was selected based on prior observation of individual datasets and a close inspection of their intensity distribution histograms. All voxels with *z* score values below this threshold were set to zero. Thus, the voxels exceeding the threshold value of z ≥ 1 were considered in our statistical analysis as having higher signal intensities (quantified as the number of voxels above a z value of 1). Mean number of voxels for each region of interest (ROI) was compared using an unpaired two-tailed *t* test (homoscedastic variances, α ≤ 0.05).

Electrophysiological data were analyzed by logistic regression (not normally distributed) or mixed model ANOVA (SAS statistic package, normally distributed) with cell identification number nested within animal identification number. Open field data were analyzed by mixed model ANOVA and adjusted for multiple comparisons using the Benjamini–Hochberg–Yekutieli false discovery rate (FDR; *p* < 0.05). Data obtained from wheel running study was analyzed by logistic regression with a negative binomial distribution. Total distances of continuous open field were analyzed by logistic regression with a gamma distribution while sleep analysis was conducted with binomial logistic regression modeling the probability of waking. Tail flick data were processed by logistic regression with a gamma distribution. GEE model in the logistic regression normalized WT or control groups in terms of current steps, wheel running, continuous open field, and tail flick to 0 without the error bar. Age and gender were used as covariates in all analysis.

To generate the hourly wheel-running activity presented in Figure 5*B*, we summed the interval counts during each hour for each animal. Wheel-running activity during the last 96 h was analyzed. Hence each animal had 4 data points for each hour. The average interval counts within each hour were calculated for each genotype. The *p* values, calculated by the unpaired Student’s test, were marked above hours in the figure. To generate the hourly probability of waking in Figure 5*I*, we determined the sleep status by the total distance traveled during 15 min in the long-term open field test as mentioned above. Therefore, there were 4 data points for each animal during each hour, which were coded from 1 and 4 as “sample.” Last 4 d’ open field activity was analyzed. Hence each animal had 4 d of data, which were coded from 1 to 4 as “period.” The probability of waking was calculated from a genotype and hour two-way interaction with repeated measurement of the period, hour, and sample using SAS logistic regression with a binomial distribution. For each genotype, SAS normalized probability of waking during each hour to the probability of the last hour either during the day or night period. The probability of waking at 6 P.M. of the control mice was set as 1, and the probabilities of the waking of other hours of the control mice were calculated relative to that of 6 P.M. To calculate the relative difference between ChKO mice and controls during each hour, we sorted the data by the hour and analyzed the probability of waking for each genotype with repeated measurement of period and sample using SAS logistic regression with a binomial distribution. The *p* values were marked above hours in the figure. The probability of waking of the ChKO mice was derived from the relative difference between the ChKO mice and the control mice.

## Results

### Increased striatal neural activity in fMRI study with the systematic *Btbd9* KO mice

**Figure 1.**
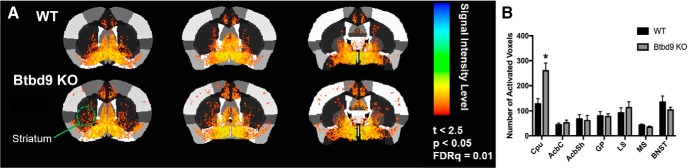
***A***, Coronal views of the averaged signal intensity from *Btbd9* KO and WT controls. ***B***, *Btbd9* KO had significant increased neural activity in caudate and putamen and in the cerebral cortex. No difference was observed in other brain regions under investigation. Bars represent means plus SEs. Cpu: caudate/putamen; AcbC: nucleus accumbens core; AcbSh: nucleus accumbens shell; GP: globus pallidus; LS: lateral septal nucleus; MS: medial septal nucleus; BNST: bed nucleus of the stria terminalis; *p < 0.05.

To study the role of the striatum in RLS pathogenesis, we first used MEMRI imaging to determine the striatal neural activity in the systematic *Btbd9* KO mice. MEMRI has been extensively used to track Ca^2+^-dependent synaptic activity (Lu et al., 2007; Hsu et al., 2008; Chiu et al., 2015; Dudek et al., 2015; Perrine et al., 2015). As a calcium analog, Mn^2+^ enters active synapses through voltage-gated calcium channels (Fukuda and Kawa, 1977; Narita et al., 1990) and is sequestered and transynaptically transported antero- and retrogradely across active neural circuits (Sloot and Gramsbergen, 1994; Pautler et al., 1998; Takeda et al., 1998a, b; Saleem et al., 2002; Murayama et al., 2006). The presence of the paramagnetic Mn^2+^ ion in the brain increases longitudinal relaxation rates and enhances signal intensity in T1 weighted scans, and is used for functional mapping of synaptic activity (Duong et al., 2000). Here, after Mn^2+^ was injected into the mice, the images of ROI were acquired (Fig. 1*A*). The result showed increased neuronal activity in the cerebral cortex of the systematic *Btbd9* KO mice, indicating an increased cortical input to the striatum. This will be addressed in detail in a separate manuscript. In the striatum, which is the focus of the current study, there was a significant increase in the caudate/putamen (Fig. 1*B*, *p* = 0.006, unpaired two-tailed *t* test), indicating increased neural activity specifically in the striatum. Increased entry of Mn^2+^ likely through voltage-gated Ca^2+^ channels also suggest an increase in Ca^2+^-dependent neural activity.

### More excitable MSNs but decreased ChI activity in the systematic *Btbd9* KO mice

**Figure 2.**
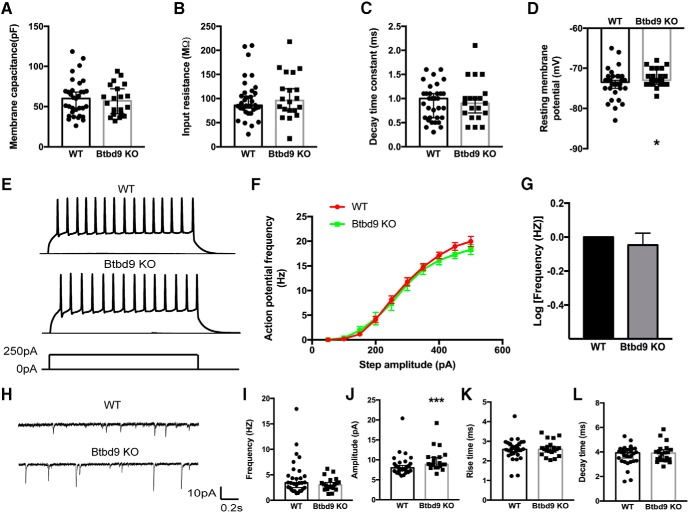
Whole-cell patch-clamp recording of MSNs from the systematic *Btbd9* KO mice and their WT littermates. ***A–C***, *Btbd9* KO MSNs (*n* = 20) did not have changes in membrane capacitance, input resistance and decay time constant compared with the WT MSNs (*n* = 33). ***D***, *Btbd9* KO MSNs (*n* = 22) had increased resting membrane potential compared with the WTs (*n* = 34). ***E***, Representative responses to the injected currents at 250 pA of KO (*n* = 21) and WT (*n* = 34) MSNs. ***F***, The frequency-current relationship for WT and *Btbd9* KO MSNs. ***G***, The response of KO MSNs to the injected currents was not significantly different from the WT MSNs. ***H***, Representative sEPSC traces of KO (*n* = 20) and WT (*n* = 30) MSNs. ***I***, The frequency of spontaneous firing was similar between the two groups. ***J***, *Btbd9* KO MSNs had a higher amplitude of sEPSC than the WT. ***K***, ***L***, Both the rise and decay time were not different between *Btbd9* homozygous KO and WT MSNs. GEE model normalized the WT group in the bar graph of ***B*** to 0 without the error bars. Data in ***A–D***, ***I–L*** were presented as median with 95% confidence intervals (CIs); ***p < 0.005, *p < 0.05.

As the first recipient for the excitatory input from almost all of the cortex, the striatum is mostly composed of MSNs (>95%; Cox and Witten, 2019). To determine the source of increased neural activity in striatum revealed by fMRI study, we did whole cell patch-clamp recording in brain slices. The result indicated that the resting membrane potential of the systematic *Btbd9* KO cells was higher than the WTs (Fig. 2*D*, *p* = 0.03, logistic regression with a gamma distribution). No change was found in membrane capacitance (Fig. 2*A*, *p* = 0.93, logistic regression with a gamma distribution), input resistance (Fig. 2*B*, *p* = 0.58, logistic regression with a gamma distribution) and decay time constant (Fig. 2*C*, *p* = 0.58, logistic regression with a gamma distribution). We then tested whether the intrinsic excitability of striatal MSNs was affected by the loss of *Btbd9*. Depolarizing current steps were injected to the MSNs of both *Btbd9* KOs and WTs (Fig. 2*E*). There was no significant difference in the frequency-current relationship (Fig. 2*F*,*G*, *p* = 0.51, logistic regression with a negative binomial distribution), amplitude, rise, and decay time (data not shown). Our results indicated that there was no change in the intrinsic excitability of the KO MSNs.

MSNs are strongly driven by glutamatergic inputs (Purves et al., 2001a). To test whether loss of BTBD9 affects excitatory synaptic transmission in MSNs, we recorded sEPSCs (Fig. 2*H*). There were no alterations in the rise time (Fig. 2*K*, *p* = 0.60, logistic regression with a gamma distribution), the decay time (Fig. 2*L*, *p* = 0.33, logistic regression with a gamma distribution), and the frequency (Fig. 2*I*, *p* = 0.11, logistic regression with a gamma distribution) of spontaneous postsynaptic currents. However, the systematic *Btbd9* KO mice showed a significantly larger amplitude of sEPSC (Fig. 2*J*, *p* = 0.004, logistic regression with a gamma distribution). The increased amplitude may be due to the increased presynaptic quantal size, increased postsynaptic functional AMPA receptor, or both. The result suggests that there are significantly enhanced excitatory inputs to the KO MSNs. In combination with the increased resting membrane potential found in the KO MSNs, the result indicates that BTBD9 deficiency may cause striatal MSNs to be more excitable.

**Figure 3.**
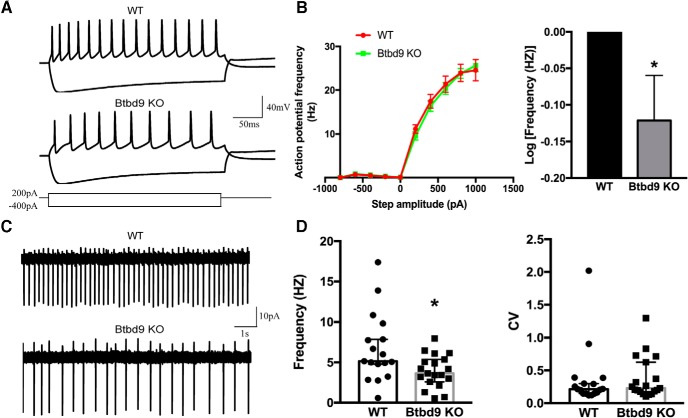
Electrophysiological recording of ChIs from the systematic *Btbd9* KO mice and their WT littermates. ***A***, Representative responses to the injected currents at 200 pA of KO (*n* = 20) and WT (*n* = 14) ChIs. ***B***, The frequency-current relationship for KO and WT ChIs. KO showed decreased firing frequency compared with the WT. WT was normalized to 0 in log transformation. ***C***, Representative traces of spontaneous firings of KO (*n* = 19) and WT (*n* = 17) ChIs. ***D***, *Btbd9* KO mice had decreased firing frequency, but no change in firing regularity in ChIs. GEE model normalized the WT group in the bar graph of ***B*** to 0 without the error bars. Data in ***D*** were presented as median with 95% CI. CV: coefficient of variance; *p < 0.05.

Abnormality in ACh neurotransmission plays an important role in movement disorders like Parkinson’s disease and dystonia (Bohnen and Albin, 2011; Dang et al., 2012; Lim et al., 2014; Eskow Jaunarajs et al., 2015). Furthermore, another RLS susceptibility gene, *MEIS1*, has been linked to the development of striatal ChIs (Spieler et al., 2014). Here, to better understand the striatal physiology in *Btdb9* KO mice and RLS, we recorded both spontaneous activity (Fig. 3*C*) and intrinsic excitability of ChIs (Fig. 3*A*). We found that ChIs of the systematic *Btbd9* KO mice had decreased intrinsic excitability (Fig. 3*B*, *p* = 0.049, logistic regression with a negative binomial distribution) and spontaneous firing activity (Fig. 3*D*, *p* = 0.04, ANOVA). It is known that MSNs inhibit the activity of ChIs through GABA and opioid receptors (Lim et al., 2014). Therefore, the alterations found in ChIs can either be a cell-autonomous effect of BTBD9 deficiency or the response to increased activity of MSNs. This was further explored in *Btbd9* ChKO mice.

### Generation and molecular characterization of *Btbd9* sKO mice

**Figure 4.**
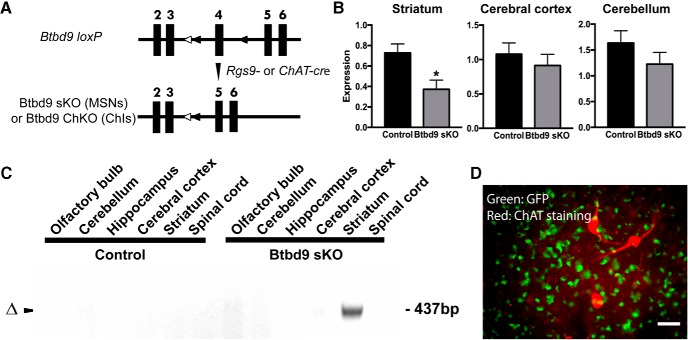
Generation of conditional KO mice and validation of the loss of *Btbd9* in the striatum by qRT-PCR. ***A***, Schematic diagram of the generation of conditional KO mice. Filled boxes represent exons. Filled triangles indicate *loxP* sites (around the 4th exon of the *Btbd9* gene). Open triangles indicate the *FRT* sites that were incorporated to remove the neo cassette. *Btbd9 loxP* mice were crossed with *Rgs9-cre* or *ChAT-cre* mice to obtain double heterozygotes. The double heterozygotes were crossed with *Btbd9 loxP* homozygotes to obtain *Btbd9* sKO or *Btbd9* ChKO mice. In conditional KO mice, exons 4 is deleted in specific types of neurons where *cre* is expressed. Recombination occurs in these cells, while other brain regions and the rest of the body still retain the intact exons. ***B***, *Btbd9* sKO mice showed a decreased level of *Btbd9* mRNA in the striatum, but not in the cerebral cortex and cerebellum. Bars represent means plus SEs; *p < 0.05. ***C***, Tissue-specific deletion of *Btbd9* exon 4 in *Btbd9* sKO mice was confirmed by PCR using DNA isolated from each brain region. The deletion was detected only in the striatum of *Btbd9* sKO mouse as predicted (Δ). ***D***, A representative immunohistochemical image of a coronal section of the striatum from an *Rgs9*-*cre*/GFP mouse. Scale bars represent 25 µm. Enlarged images captured with a 40× objective lens showed that the ChAT staining (red) did not overlap with GFP staining (green). The results suggest that *Rgs9-cre* does not have cre-mediated recombination in ChIs.

To further study the critical role of the striatum in RLS, we generated two conditional KO mouse models in which the *Btbd9* gene was selectively knocked out either in the MSNs or ChIs (Fig. 4*A*). Specifically, we interbred *Rgs9-cre* mice with *Btbd9 loxP* mice to obtain *Btbd9* sKO mice. The transcription of *Btbd9* gene was quantified by qRT-PCR. As expected, there was a significant reduction of *Btbd9* mRNA in the striatum compared to control littermates (Fig. 4*B*, *p* = 0.047, paired two-tailed *t* test), but not in the cerebral cortex or cerebellum (Fig. 4*B*, cerebral cortex, *p* = 0.51; cerebellum, *p* = 0.28; both paired two-tailed *t* test). To future confirm tissue specificity of the KO, we dissected out different brain regions from the *Btbd9* sKO mice and their controls. DNAs were extracted from these different brain regions, and PCR reactions were conducted. Only the DNA extracted from the striatum of *Btbd9* sKO mice showed the recombined band (Fig. 4*C*), indicating that the KO was restricted to the striatum. It is worth noting that the remaining *Btbd9* expression in the striatum can be accounted for by the small subset of interneurons (<4%) that do not express *cre*, and non-neuronal cells such as glial cells. To determine whether ChIs express *Rgs9*-*cre*, we crossed *Rgs9*-*cre* mice with GFP indicator mice and generated *Rgs9*-*cre*-GFP mice. There were no overlaps between 100 randomly selected, ChAT-positive neurons with any of the GFP-positive neurons (Fig. 4*D*). The results suggest that *Rgs9-cre* does not induce gene recombination in ChIs and *Btbd9* gene is mostly knocked out in the MSNs of *Btbd9* sKO mice.

### RLS-like phenotypes in *Btbd9* sKO but not in *Btbd9* ChKO mice

**Figure 5.**
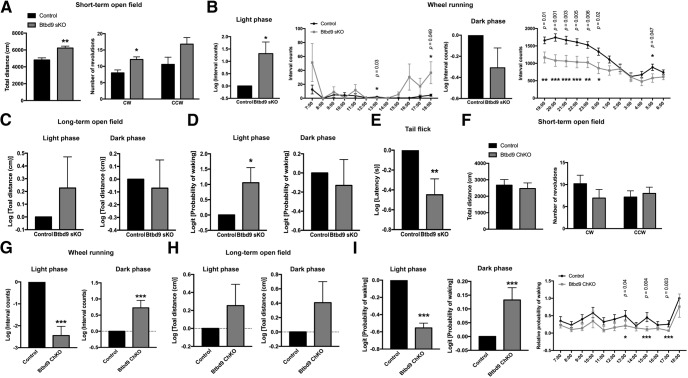
Behavior studies of the conditional KO mice. ***A***, *Btbd9* sKO mice traveled more and showed an increase in CW circling in the 30-min open field test. ***B***, *Btbd9* sKO mice had increased activity in the wheel running test compared with the controls during the light phase, but not in the dark phase. Detailed hourly differences are listed (see Materials and Methods). Significant *p* values are marked above the corresponding time points. ***C***, In the long-term open field test, *Btbd9* sKO mice did not show a significant change in the total distance traveled during both the light and the dark phases. ***D***, *Btbd9* sKO showed an increased probability of waking in the light phase of the long-term open field test. ***E***, *Btbd9* sKO mice showed shorter latencies to respond to a warm stimulus in the tail flick test. ***F***, *Btbd9* ChKO mice did not show activity alteration in the 30-min open field test. ***G***, *Btbd9* ChKO mice ran less during the light phase but ran more during the dark phase in the wheel running test. ***H***, In the long-term open field test, *Btbd9* ChKO mice did not have a significant change in the total distance traveled during both the light and the dark phases. ***I***, The probability of waking significantly decreased during the light phase and significantly increased during the dark phase for the *Btbd9* ChKO mice. Detailed hourly differences are listed (see Materials and Methods). Significant *p* values are marked above the corresponding time points. GEE model normalized the WT or control group in the bar graphs of ***B–E***, ***G–I*** to 0 without error bars. Bars represent means plus SEs; ***p < 0.005, **p < 0.01, *p < 0.05.

A principal feature of RLS is a desire to move (Ferré et al., 2019). Previous mouse or fruit fly models of RLS have shown increased activity levels (DeAndrade et al., 2012a; Freeman et al., 2012). Therefore, we used an open field activity chamber to assess the total activity levels of the *Btbd9* sKO and *Btbd9* ChKO mice. In the short-term 30-min open field test, we observed that although there was no alteration found with *Btbd9* ChKO mice [Fig. 5*F*, total distance, *p* = 0.69; clockwise (CW), *p* = 0.29; counter-CW (CCW), *p* = 0.70; all ANOVA], *Btbd9* sKO mice exhibited significantly increased total distance traveled (Fig. 5*A*, total distance, adjusted *p* = 0.01, ANOVA) and vertical activity (Table 1, adjusted *p* = 0.049, ANOVA) compared with control mice. Vertical activity here represents rearing behavior (Tatem et al., 2014). Furthermore, *Btbd9* sKO mice had a significant increase in CW circling, while there was no statistical difference in CCW circling compared with control mice (Fig. 5*A*, CW, adjusted *p* = 0.02, ANOVA; CCW, adjusted *p* = 0.095, ANOVA). Finally, there were no significant differences in stereotypical behavior or anxiety in the mice (Table 1). The increased activity level suggests that *Btbd9* sKO mice are hyperactive. Furthermore, alterations in circling behaviors indicate imbalances in the striatal dopaminergic system (Fornaguera and Schwarting, 2002). In the long-term open field test, *Btbd9* sKO mice showed no change (Fig. 5*C*, left panel, *p* = 0.35, logistic regression with a gamma distribution) in the total distance traveled in the light phase, when mice are usually sleeping or resting. However, the sleep analysis indicates that the probability of waking of *Btbd9* sKO mice significantly increased in the light phase (Fig. 5*D*, left panel, *p* = 0.03, logistic regression with a binomial distribution), but did not change in the dark phase, when mice are usually active (Fig. 5*D*, right panel, *p* = 0.63, logistic regression with a binomial distribution). The symptoms of RLS patients usually occur or become worse in the evening or at night (Garcia Borreguero et al., 2017). With opposite day-night rhythms to human, *Btbd9* sKO mice had the motor restlessness with a similar circadian predominance as patients. In contrast, although *Btbd9* ChKO mice did not show any difference in total distance traveled compared with the controls (Fig. 5*H*, left panel, *p* = 0.28; right panel, *p* = 0.16; both logistic regression with a gamma distribution), they had a decreased probability of waking during the light phase (Fig. 5*I*, left panel, *p* < 0.0001, logistic regression with a binomial distribution), especially at 1, 3, and 5 P.M. (Fig. 5*I*, right panel), and increased probability of waking during the dark phase (Fig. 5*I*, middle panel, *p* = 0.0029, logistic regression with a binomial distribution), suggesting that the animals sleep better than their controls. It should also be noticed that the increased probability of waking found in *Btbd9* sKO mice mainly appeared during the second half of the rest phase (data not shown), which is consistence with clinical observations.

**Table 1.** Higher level of vertical activity and no alteration in anxiety or stereotypical behaviors in the open field

Genotype	Control (*n* = 7)	*Btbd9* sKO (*n* = 8)	*p* value	Adjusted *p* value
Vertical activity	410 ± 48	580 ± 45	0.026	0.049*
Center time	328 ± 43	370 ± 41	0.50	0.6
Center distance/total distance	0.27 ± 0.01	0.29 ± 0.01	0.43	0.5
Stereotypy count	2071 ± 120	2308 ± 112	0.18	0.3

Vertical activity is presented as the mean number of beam breaks ± SEs. Center time is presented in seconds. Stereotypy count is presented as the number of counts; *p* values have been adjusted for multiple comparisons using the Benjamini–Hochberg–Yekutieli FDR (*p* < 0.05).

Next, we conducted a wheel running study to measure the voluntary activity of these mice under the normal 12 LD condition. *Btbd9* sKO mice showed a significantly elevated level of activity compared with controls during the light and rest phase (Fig. 5*B*, light phase, *p* = 0.0004, logistic regression with a negative binomial distribution), but a similar level of activity as control mice during the dark and active phase (Fig. 5*B*, dark phase, *p* = 0.32, logistic regression with a negative binomial distribution). On the other hand, *Btbd9* ChKO mice showed a significantly decreased activity level during the light and rest phase (Fig. 5*G*, left panel, *p* < 0.0001, logistic regression with a negative binomial distribution) but increased activity level during the dark and active phase (Fig. 5*G*, right panel, *p* = 0.0015, logistic regression with a negative binomial distribution). These data are consistent with the long-term open field test and suggest that only *Btbd9* sKO mice have an increase in voluntary activity during their rest period. The increased voluntary activity mainly appeared during the second half of the rest phase and the first half of the active phase. Taken together, both total activity and voluntary activity were increased in the *Btbd9* sKO mice in the rest phase, which resembles aspects of nocturnal RLS activity found in patients. The circadian component-involved behavior of *Btbd9* ChKO mice is completely contradictory to *Btbd9* sKO mice. Loss of BTBD9 protein only in MSNs, but not ChIs, can cause diurnal motor restlessness in mice.

Uncomfortable sensations in lower limbs are another common phenotype of RLS (Garcia Borreguero et al., 2017). Therefore, we tested the *Btbd9* sKO mice for abnormalities in the sensory system using the tail-flick test. The mutant mice had a higher level of sensitivity to the heat stimuli (Fig. 5*E*, *p* = 0.005, logistic regression with a gamma distribution), indicating that mice lacking BTBD9 specifically in MSNs developed alterations in thermal sensation as the systematic *Btbd9* KO.

### Increased ChIs excitability in *Btbd9* ChKO mice

**Figure 6.**
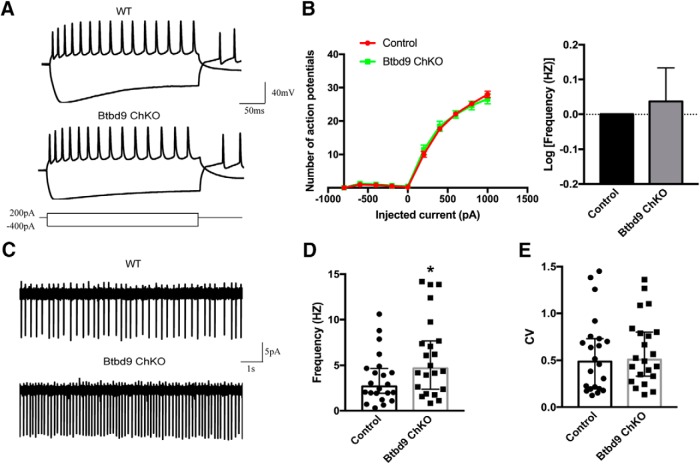
Electrophysiological recording of ChIs from *Btbd9* ChKO mice and their control littermates. ***A***, Representative responses to the injected currents at 200 pA of ChKO (*n* = 16 cells) and control (*n* = 18 cells) mice. ***B***, The frequency-current relationship for ChKO and control ChIs. Firing frequency of mutant ChIs did not change compared with the controls. ***C***, Representative traces of spontaneous firings of mutant (*n* = 22) and control (*n* = 22) ChIs. ***D***, ChKO mice had increased ChIs firing frequency. ***E***, Firing regularity of ChIs was not different between mutants and controls. GEE model normalized the control group in the bar graph of ***B*** to 0 without the error bars. Data in ***D*** were presented as median with 95% CI. CV: coefficient of variance; *p < 0.05.

The behavioral data showed that BTBD9 deficiency in MSNs, but not in ChIs, can lead to RLS-like phenotypes. Therefore, it is likely that the KO of *Btbd9* in ChIs alone did not contribute directly to the behavioral and electrophysiological alterations observed in the systematic *Btbd9* KO mice. To explore this, we recorded the ChIs in *Btbd9* ChKO mice for both spontaneous activity (Fig. 6*C*) and responses to the injected currents (Fig. 6*A*). We did not find the decreased activity of ChIs in *Btbd9* ChKO mice, as observed in the systematic *Btbd9* KO (Fig. 3). Instead, spontaneous activity of ChIs in the mutant mice was significantly increased (Fig. 6*D*, *p* = 0.02; 6*E*, *p* = 0.74; all ANOVA), although there was no change found in intrinsic excitability (Fig. 6*B*, *p* = 0.70, logistic regression with a negative binomial distribution). Hence, a lack of *Btbd9* in ChIs alone led to elevated ChI activity.

## Discussion

In this study, we determined how the loss of *Btbd9* affects striatal physiology and focused on the role of MSNs in RLS pathogenesis. Using both brain imaging *in vivo* and electrophysiological recording *in vitro*, we found that the systematic *Btbd9* KO mice had enhanced neural activity in the striatum, more excitable MSNs, and decreased activity in the ChIs. In addition, specific loss of *Btbd9* in the MSNs was sufficient to cause RLS-like phenotypes. When *Btbd9* was conditionally knocked out in the ChIs, the mice showed neither RLS-like behavioral phenotypes or maintained decreased activity in the ChIs as observed with the systematic *Btbd9* KO mice. The results suggest that activity changes in the ChIs in the systematic *Btbd9* KO mice are not cell-autonomous but result from alteration of striatal circuits, including changes in the MSNs. It should be noted that the current study is not aimed at generating new animal models for RLS. Instead, our findings demonstrate that striatum, especially MSNs, is critically involved in the development of RLS-like phenotypes in mice.

Our imaging study revealed increased neural activity in the systematic *Btbd9* KO striatum. Close to 95% of cells in the striatum are MSNs. Therefore, the result suggests a possible overall increase in the Ca^2+^-dependent neural activity of MSNs. Furthermore, electrophysiological results showed an increased excitatory synaptic transmission onto striatal MSNs while their intrinsic neuronal properties were not altered. No change in the frequency, but the higher amplitude of sEPSC suggest that the MSNs of the KO mice seem to have increased excitatory synaptic inputs. Combined, our data suggest an enhancement of corticostriatal or thalamostriatal synaptic activity. Similarly, in iron deprived rat, which is thought to be an RLS rodent model, corticostriatal excitability is elevated (Yepes et al., 2017). Here, it is not known whether inputs from corticostriatal, thalamostriatal, or both contributed to the increased striatal sEPSC amplitude in the systematic *Btbd9* KO mice. Differential alterations of thalamostriatal and corticostriatal synapses have been found in mouse models of Huntington's disease (Deng et al., 2014; Kolodziejczyk and Raymond, 2016; Parievsky et al., 2017), an MPTP-treated monkey model of parkinsonism (Raju et al., 2008) and a rat model with L-DOPA-induced dyskinesias (Zhang et al., 2013). Future studies will be focused on dissecting the differential effects of the systematic *Btbd9* KO on corticostriatal and thalamostriatal synaptic transmission.

The electrophysiological recording revealed a decreased excitability in striatal ChIs in the systematic *Btbd9* KO mice. It has been found that *Gbx2*, a gene which is essential for the proper development of striatal ChIs, is downed-regulated in heterozygous *Meis1* KO embryos (E12.5; Spieler et al., 2014). *MEIS1* is another gene implicated in RLS (Schormair et al., 2017). With conditional KO mice and electrophysiological recordings, we found that like *Meis1* mice, *Btbd9* mutation in the ChIs alone can cause functional abnormalities in striatal ChIs, yet it was insufficient to produce RLS-like behaviors. In the rest phase, *Btbd9* ChKO mice had decreased locomotor activity with increased excitability of striatal ChIs, while the systematic *Btbd9* KO mice showed increased locomotor activity (DeAndrade et al., 2012a) with decreased excitability in striatal ChIs. The results suggest a critical role of ChIs in movement control in the rest phase, and the decreased ChI activity found in the systematic *Btbd9* KO mice is not a cell-autonomous effect. It is known that ACh can regulate striatal circuit through its receptors on MSNs, GABAergic interneurons, glutamatergic and DA terminals (Lim et al., 2014). It is possible that excess release of ACh in *Btbd9* ChKO mice downregulated activities of MSNs directly through the nAChRs (Liu et al., 2007) and M4 receptors (Howe and Surmeier, 1995) or indirectly by increasing GABAergic inhibition on MSNs (English et al., 2011). As the sole output of the striatum, MSNs may lead to decreased locomotion through their decreased activities. However, when *Btbd9* was knocked out systematically, neuronal activities were changed in both MSNs and ChIs. Our results showed that MSNs became more excitable and may have increased activity. These changes in MSNs were likely to be dominant and overcame the influence of ChIs. MSNs, via their inhibition on the ChIs, in turn, led to decreased activity in ChIs as showed by the electrophysiological recording. Although it is not clear if the activity level of MSNs in the *Btbd9* sKO mice is the same as what we found in the systematic *Btbd9* KO, *Btbd9* sKO mice did show an opposite output in behavioral tests as *Btbd9* ChKOs, which support the overwhelmingly inhibitory effect of MSNs to ChIs as mentioned above.

MSNs-specific *Btbd9* KO was found to be sufficient to induce rest-phase specific hyperactive movement, sleep disturbance, and increased thermal sensation in mice. The findings support the idea that striatum is critical for the pathogenesis of RLS motor phenotypes. Postmortem studies comparing RLS patients and control group show a decreased D_2_R expression in the putamen, but increased phosphorylated tyrosine hydroxylase (TH), a rate-limiting enzyme for DA synthesis, in both putamen and SN (Connor et al., 2009). Brain imaging studies indicate decreased membrane-bound DAT level and D_2_R binding potential in the striatum (Rizzo et al., 2017). Additionally, changes in iron hemostasis have been found in SN and putamen of RLS patients (Allen et al., 2001; Earley et al., 2014). Striata of iron-deprived RLS rodent models show a reduced density of DAT and DA receptors (Erikson et al., 2000, 2001) and enhanced release at corticostriatal terminals (Yepes et al., 2017). Taken together, these studies suggest functional alterations in the striatum are correlated with RLS (Rizzo et al., 2017). In addition, a recent GWAS study demonstrated that striatal MSNs are associated with insomnia (Jansen et al., 2019), which can be caused by RLS.

However, dysfunctional striatal circuit caused by the loss of BTBD9 might not be the only mechanism in the systematic *Btbd9* KO mice. *Btbd9* is also expressed in the spinal cord, although our genomic PCR failed to detect any deletion of the *Btbd9* gene in the spinal cord of the *Btbd9* sKO mice. Alterations in the striatum can lead to alterations in the spinal cord. The basal ganglia output modulates the spinal cord through feedback to the cortex. Additionally, recent evidence has suggested that a microcircuit exists between the corticostriatal tract and the corticospinal tract, starting in the striatum and ending in the spinal cord (Kiritani et al., 2012). Spinal neural circuits have been proposed to be central in RLS development (Clemens et al., 2006; Zwartbol et al., 2013; Koblinger et al., 2014; Kumru et al., 2015). It has been found that a lesion in a dorsoposterior hypothalamic dopaminergic A11 cell group, which is the sole source of spinal DA, leads to increased wakefulness across the rest phase (Ondo et al., 2000) and a long-lasting reduction in sensory thresholds (Clemens et al., 2006) in rats. D_3_ receptors are mostly present in the dorsal spinal cord where it has been shown to modulate sensory pathways (Meneely et al., 2018). Mice knocked out of D_3_ receptors (D_3_KO) show increased locomotion (Accili et al., 1996; Clemens et al., 2006) and decreased paw withdrawal latencies to thermal pain stimulation. Furthermore, D_3_KO mice have a reduction in frequency-dependent modulation of the longer-latency reflex (LLRs) in the spinal cord (Keeler et al., 2012). Pharmacological experiments indicate that D_3_KO mice exhibit a reversal of the modulatory actions of DA on spinal reflexes from depression to facilitation (Clemens and Hochman, 2004), and are responsive to D_1_ and D_2_ receptor agonists (Keeler et al., 2012). These findings emphasize the role of spinal DA in the etiology of RLS.

Our results provide a novel mechanism for the efficacy of dopaminergic agonists as treatments for RLS. The dopaminergic system plays a critical role in RLS pathogenesis. In addition to the evidence mentioned above, RLS patients have upregulated levels of L-DOPA metabolites and TH activity in CSF (Earley et al., 2001; Allen et al., 2009). RNAi-mediated knock-down of *BTBD9* homolog gene in a subset of dopaminergic neurons can reproduce sleep phenotype in fruit flies (Freeman et al., 2012). Ropinirole rescues the sensory deficit found in the systematic *Btbd9* KO mice (DeAndrade et al., 2012a). It is possible that increased activity of MSNs leads to a decreased firing in the ChIs and a lower level of ACh. Nigrostriatal DA terminals express both ionotropic nicotinic ACh (nACh) and G_q/11_-coupled muscarinic ACh (mACh) receptors (Cachope and Cheer, 2014). The activation of nACh receptors elicits DA release, while the downstream pathway of mACh receptor inhibits DA release. Overall, optogenetic stimulation of ChIs evokes DA release in slice preparation (Cachope et al., 2012; Cachope and Cheer, 2014). Therefore, a decreased level of ACh may cause a deficiency in DA release. It has also been found that activation of MSNs through AMPA receptor leads to the generation of a diffusible messenger, hydrogen peroxide (H_2_O_2_), which can inhibit DA release via ATP-sensitive potassium channels (Avshalumov et al., 2008; Sulzer et al., 2017). Either way, increased MSNs activity is predicted to reduce striatal DA release in the systematic *Btbd9* KO mouse model, which is consistent with the clinical finding that DA agonists can be used to treat RLS.

In summary, our results suggest that alteration in the striatal circuit, especially increased activity of the striatal MSNs, could potentially serve as a main pathogenetic mechanism of the motor and sensory dysfunction in RLS. It also supports an indirect role of the striatal ChIs in the disease development. Finally, these data present a plausible explanation for the therapeutic efficacy of the DA receptor agonists in RLS that includes the inhibition of the MSNs activity, the regulation of ChI excitability, and the resetting of the striatal circuit. Further investigations are needed to dissect the complex interactions among the striatal neurons and modulatory neurotransmitters, which will aid the development of highly selective anti-RLS drugs with fewer side effects.

## Synthesis

Reviewing Editor: Kirill Martemyanov, The Scripps Research Institute Decisions are customarily a result of the Reviewing Editor and the peer reviewers coming together and discussing their recommendations until a consensus is reached. When revisions are invited, a fact-based synthesis statement explaining their decision and outlining what is needed to prepare a revision will be listed below. The following reviewer(s) agreed to reveal their identity: Nandakumar Narayanan. Note: If this manuscript was transferred from JNeurosci and a decision was made to accept the manuscript without peer review, a brief statement to this effect will instead be what is listed below.

I would include the raw open-field data as the video is not available.

I find the manuscript substantially improved. Major comments:

1) I'm still not sure what to make of the MEMRI data - and that it a faithful index of neuronal activity. Are authors' data similar to prior work? It would seem critical to cite this work here.

2) I would point out and discuss that this might not be the only mechanism of Btbd9 function, that it is expressed in the spinal cord, and include a paragraph on the discussion on this point.
